# AI through the looking glass: an empirical study of structural social and ethical challenges in AI

**DOI:** 10.1007/s00146-024-02146-0

**Published:** 2024-12-15

**Authors:** Mark Ryan, Nina de Roo, Hao Wang, Vincent Blok, Can Atik

**Affiliations:** 1 Wageningen Economic Research, Wageningen University and Research, Droevendaalsesteeg 4, 6708 PB Wageningen, The Netherlands; 2 Philosophy Group, Wageningen University and Research, P.O. Box 8130, 6700 EW Wageningen, The Netherlands

**Keywords:** Artificial intelligence, Agriculture, Agri-food, AI ethics, Structural challenges, Data policy

## Abstract

This paper examines how professionals (N = 32) working on artificial intelligence (AI) view structural AI ethics challenges like injustices and inequalities beyond individual agents' direct intention and control. This paper answers the research question: What are professionals’ perceptions of the structural challenges of AI (in the agri-food sector)? This empirical paper shows that it is essential to broaden the scope of ethics of AI beyond micro- and meso-levels. While ethics guidelines and AI ethics often focus on the responsibility of designers and the competencies and skills of designers to take this responsibility, our results show that many structural challenges are beyond their reach. This result means that while ethics guidelines and AI ethics frameworks are helpful, there is a risk that they overlook more complicated, nuanced, and intersected structural challenges. In addition, it highlights the need to include diverse stakeholders, such as quadruple helix (QH) participants, in discussions around AI ethics rather than solely focusing on the obligations of AI developers and companies. Overall, this paper demonstrates that addressing structural challenges in AI is challenging and requires an approach that considers four requirements: (1) multi-level, (2) multi-faceted, (3) interdisciplinary, and (4) polycentric governance.

## Introduction

Artificial intelligence (AI) has received much public attention in the past couple of decades, particularly for the ethical and societal risks it raises. There have been concerns about what kind of safeguards and control measures are in place to ensure that society benefits from AI without suffering the threats that may arise from its (mis)use. There is a growing call to identify and find solutions to these challenges, often in the form of ‘AI ethics guidelines’ (for example, Corrêa et al. 2023, identify at least 200 guidelines). Additionally, AI ethics is emerging as a field of research in its own right, with over 100 different frameworks and approaches emerging to tackle the challenges AI poses (e.g., value-sensitive design (VSD), ethics by design (EbD), and ethical, legal, and social aspects (ELSA)) (see Prem 2023).

These guidelines and frameworks offer the potential to redesign AI and provide recommendations to AI developers and companies. While the goals of these approaches are praiseworthy, the scope of analysis may sometimes be too narrow, and as a result, specific challenges may get overlooked. AI ethics guidelines and frameworks often give recommendations to individuals or companies developing AI, so they need to be practical and actionable. A disadvantage of these approaches is that they often do not tackle many AI-related issues beyond the scope of implementability by AI developers or the companies they work for. Furthermore, these recommendations are often in stark contrast with issues that the public is most concerned about. Public concerns are often related to much broader social phenomena that go beyond the capacity of AI developers and tech companies (e.g., some of the most pressing issues discussed in a recent PEWS Research report are the lack of human connection, surveillance and loss of jobs as a result of AI) (Rainie et al. 2022).

These broader issues may not necessarily relate to or can be solved by approaches focusing on ethics guidelines alone. As a result, there is the potential that many ethics guidelines and approaches will lose sight of these challenges by only focusing on actions that are more implementable and achievable by their target audience (i.e., developers and AI companies). For example, surveillance is not only caused by the design of AI or by not following an ethics guideline correctly but also because of multiple, large-scale processes, political agendas, and societal and historical relationships (Zuboff 2019). Ethics guidelines and frameworks are much less suitable for analyzing these types of ‘structural challenges’, which may be caused or exacerbated by AI. Structural challenges refer to challenges caused by patterned inequalities, often produced and reproduced through multiple, large-scale social processes where no identifiable agent directs, controls, or intends (Haslanger 2023).

While structural challenges are sometimes discussed in the literature, they are often described in abstract terms (e.g., pan-surveillance (Zuboff 2019), objectification (Wang 2022), pan-computationalism (Nadin 2013; Mau 2019), and commodification (Preston 2022; Roessler 2015; Seidl 2023).^1^ Additionally, structural challenges are complex and nuanced; addressing them requires the involvement of many different stakeholders in myriad ways. This may explain why it is difficult to encapsulate them in ethics guidelines or AI ethics frameworks. While many AI approaches focus on ethics guidelines and building values into AI (see Prem 2023), we want to expand the debate to include structural challenges. This research empirically explores how professionals working on AI (N = 32) view structural challenges.^2^ The primary research question of this paper is: What are professionals’ perceptions of the structural challenges of AI (in the agri-food sector)?

Concentrating on a sector-level application of AI (e.g., agri-food) was to gather nuances about how structural challenges are felt in a specific context (rather than evaluating AI in a too generic way). This paper evaluates the results from four multi-stakeholder workshops to concentrate on the application of AI in the agri-food sector.

Section 2 of this paper consists of a preliminary review of the literature on the structural challenges of AI and some typical responses to address them. This review provides the theoretical underpinnings for our empirical research on how these structural challenges are felt within agri-food. Section 3 describes the methodology we employed in this study. This section is followed by the results from the workshops (Sect. 4) and a discussion on how the identified structural challenges can be addressed within the AI ethics debate (Sect. 5).

Our paper concludes that requiring AI developers to encode values into AI systems (alone) is insufficient. While it is undoubtedly a fundamental step in the right direction, it is an insufficient approach to address the structural issues identified in this paper. Instead, addressing structural challenges in AI requires a ‘multi-level’ (micro, meso, and macro) (Sect. 5.1.), multi-faceted (QH stakeholders) (Sect. 5.2.), interdisciplinary (Sect. 5.3.), and a polycentric governance approach (Sect. 5.4.).

## Literature review

The term 'Artificial Intelligence (AI)' emerged in 1956 during the Dartmouth Conference when John McCarthy and colleagues coined it, envisioning machines that could mimic human intelligence (Kaplan & Haenlein 2019). AI is often seen as a system, or a set of systems, that can exhibit intelligent behavior by analyzing their surroundings and autonomously taking actions to complete specific objectives (European Commission 2018).^3^

For decades, limited computational power largely restrained AI’s advancement. However, in the late 2010s, there was a growth in AI development (known as the 'AI boom' or 'AI spring'), thanks to the breakthroughs in machine learning, neural networks, and increased computational capabilities (Bommasani, Rishi 2023). AI has gained significant investment and interest by promising efficiency, reducing costs, and solving global challenges (e.g., poverty and climate change). Today, AI is increasingly becoming an integral part of our daily lives, and it is used in various applications, from voice assistants and recommendation systems to autonomous vehicles and advanced medical diagnostics (OECD 2022).

However, the wide use of AI is profoundly shaping many crucial parts of our world, posing ethical, legal and societal concerns. For instance, poorly designed AI systems may result in biased results and privacy concerns (Leslie 2019). The deployment of AI can also result in diminishing human oversight and compromising individual autonomy (Tsamados et al., 2020). Other authors point to the risk that AI can potentially erode democracy in political spheres through disinformation and manipulating end-users decision-making capabilities (Susser et al. 2019; Zuboff 2019; Sax 2021). Moreover, the automated nature of AI triggers the fear of job loss and unemployment, creating a sense of insecurity for workers (Acemoglu et al. 2018; Rawashdeh 2023). Generative AI can lead to violations of intellectual property rights (Wach et al. 2023). The ‘black box’ nature of AI makes it challenging to identify the cause of these issues and who is responsible for them (Franke 2022; Wang 2022).

### AI ethics guidelines and approaches to AI

In response, a surge of literature on AI ethics and governance underscores the importance of ensuring that AI benefits humanity while minimizing potential risks and harms. The most common approach to the challenges faced in AI in recent years is the establishment of AI ethics guidelines (Jobin et al. 2019). AlgorithmWatch (2020) compiled an inventory of over 160 AI ethics guidelines, most of which emerged after 2018 and highlighted the need to develop responsible and trustworthy AI (e.g., AI HLEG 2019, 2020, and AIEIG 2020).^4^ They are often created by the public (e.g., the OECD, the European Commission High-level Expert Group, 2019, 2020, and IEEE 2020, 2021) or private organizations (Google, Microsoft, and DeepMind) (de Laat 2021). These guidelines are typically aimed at engineers or organizations developing AI. They represent a top-down method of addressing values and regulating AI risks (Sadek et al. 2023; Umbrello and van de Poel 2021).

Besides ethics guidelines, several emerging frameworks start ‘with high-level values for AI, which are then translated into design requirements for AI systems, further translated into specific measures to be undertaken at specific points in the design process’ (Brey & Dainow 2023, 11). This framework operationalizes ethical values and principles by providing engineers with tangible tasks to be completed throughout the AI system’s entire lifecycle. An example is the 'Ethics by Design' approach, which is gaining popularity in AI and is seen in similar approaches, such as Privacy by Design, Transparency by Design, and Sustainability by Design (Schaar 2010; Felzmann et al. 2020).

Another approach to the challenges faced in AI is developed through Value-Sensitive Design (VSD) (Sadek et al. 2023). The basic idea of VSD is to include stakeholders’ values and preferences that are not necessarily derived from high-level guidelines in design decisions from the start and throughout the process (Friedman et al. 2013; Umbrello and van de Poel 2021). It starts with stakeholders’ values relevant to a particular application of AI, such as autonomous lethal weapons (Umbrello 2019a; Boshuijzen-van Burken et al. 2023), worker assistance systems (Vernima et al. 2022), smart cities (Helbing et al. 2021), nanotechnology (Umbrello 2019b), and care robots (van Wynsberghe 2020).

Other approaches include the Ethical Legal and Societal Aspects (ELSA) Lab's approach (NLAIC 2022; Ryan & Blok 2023). This approach originated from the literature on ELSA (Zwart and Nelis 2009) and was developed after the Netherlands dedicated approximately 10 million euros to these AI ELSA labs (Veenstra et al. 2021). By 2023, 23 AI ELSA Labs have been established nationwide, with expectations for more in the coming years, each focusing on a specific area of AI application. An essential feature of ELSA Labs’ approach involves fostering a collaborative environment where academia, industry, government, and civil society join forces to make AI 'ethical in practice' (NL AIC 2021, 2023).

### Limitations of ethics guidelines and EbD, VSD, and ELSA

Many ethical challenges often arise from the design, development, or implementation of AI technology (Dignum et al. 2018; Brey & Dainow 2023; Sanderson et al. 2023). Therefore, approaches like EbD, VSD, and ELSA Lab may be helpful in responding to concerns about data collection, algorithm processing, and the content produced by AI applications (OECD 2022; Mökander et al. 2023). However, there is a tendency to focus on AI guidelines and recommendations that can be ethically designed or developed by engineers and programmers, and any challenges can be easily fixable and solvable in the design process. Ethics guidelines and approaches like VSD, EbD, and ELSA often concentrate on what the engineer must do to program values into the AI system.

Because ethics guidelines and approaches like EbD, VSD, and ELSA also typically have a very particular focus (e.g., on ethics, values, etc.), there is the possibility they overlook other areas (such as economic, technological, and governance issues) (Ryan & Blok 2023). The focus of all three approaches EbD, VSD, and ELSA) is on micro-analysis ('case studies'), which inhibits them from examining larger socio-economic issues (Blok 2024; Zwart et al. 2014, p. 10). As a result, structural challenges may get overlooked with such a narrow focus on the obligations of engineers or companies to develop more ‘trustworthy’, ‘responsible’, or ‘value-sensitive’ AI.

Ethics guidelines, VSD, EbD, and ELSA risk oversimplifying complex structural challenges that need multi-stakeholder engagement and broader interdisciplinary actions (Hagendorff 2022; Sadek et al. 2023). Take fairness as an example, it’s frequently associated with and centered on data and algorithms, flattening the understanding of fairness to a ‘purely objective’ dataset (Benjamin 2019; Birhane 2021). Addressing fairness is followed by attempts to solve these problems with straightforward programming (e.g., debiasing datasets). However, this process overlooks the option that the bias is caused by race, gender, intersectional or other historical inequalities. These structural issues are not necessarily easily fixable by optimizing one’s algorithm.

### The need to examine structural challenges of AI

**Fig. 1:**
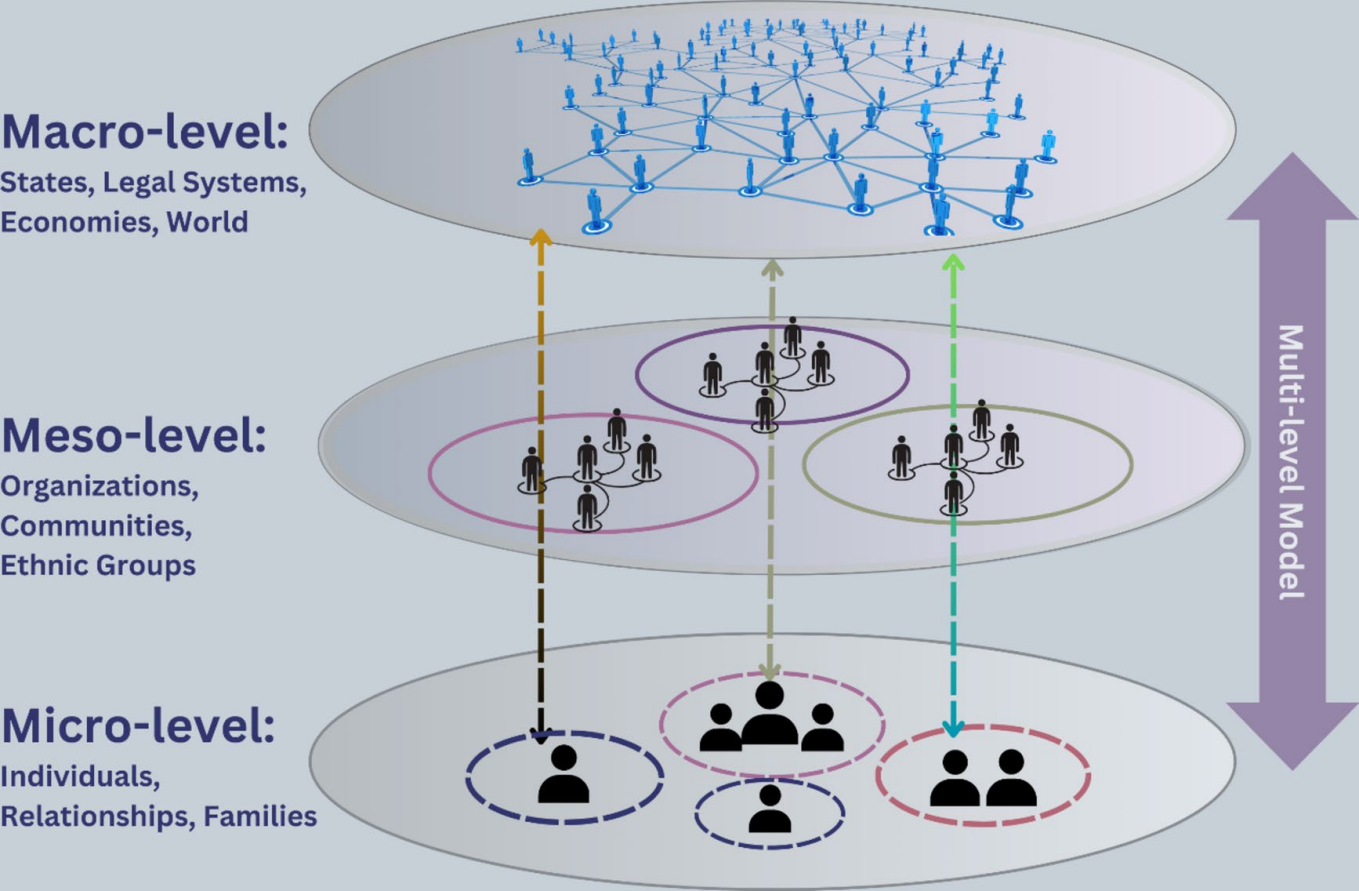
Micro-, meso-, and macro-levels of society

While structural issues may emerge through relationships and interactions with others and from varying organizational structures, they originate from deeply embedded policies of states, legal systems, and entire economies (also known as macro-levels of society) (see Fig. 1).

Structural challenges are not created by an individual but stem from broader social–political structures and historical inequalities (Young 2001). Understanding structural challenges involves more than recognizing isolated bias or privacy issues; it requires delving into the mechanisms to disclose how these individual challenges can intertwine with broader social structures. Structural challenges can cause different forms of harm and oppression that impact individuals (micro-level) and groups, typically vulnerable and marginalized communities (meso-level). Structural harms include, for instance, the maintaining and even strengthening of existing unequal social and cultural structures (Hagendorff 2022; Sadek et al. 2023).

To illustrate this point, we will use the example of an application of AI within the agri-food sector: an AI-based recommendation system that gives farmers personalized suggestions on crop selection, disease treatment, and pest control (Ryan et al. 2023). Personalized recommendations are generated by AI systems that gather and calculate historical data on farming practices on various farms. However, historically, these farming practices can be structurally different in large-scale farms and smallholders. Large farms owned by big companies often highlight efficiency and maximizing profit-making, dominated by some industrialized farming practices like monocropping, automation, synthetic fertilizers and pesticides (Ditzler & Driessen 2022). By contrast, smallholders, especially those who believe in agroecology, may not embrace these efficiency-focused approaches but may adopt some methods that prioritize community connection and eco-friendliness, such as diversified cropping systems, organic practices, or agroecological approaches (Wezel et al. 2020). Suppose historical data are collected mainly from large-scale farms; the AI algorithms may be trained in a way that only favors and promotes industrialized farming practices while marginalizing those employed by smallholders. This algorithm may mirror and perpetuate structural inequalities in the agri-food system.

This paper examines how professionals in the agri-food sector perceive structural challenges and solutions concerning AI. This research also identifies at what level of societal analysis those in the field think about ethical challenges and solutions. It is important to understand common structural challenges of AI in agri-food, why they are considered significant, and identify recommendations on how to address them. This paper provides a foundation for future endeavors to integrate structural reflection into practical AI discussions.

## Methodology

**Fig. 2:**
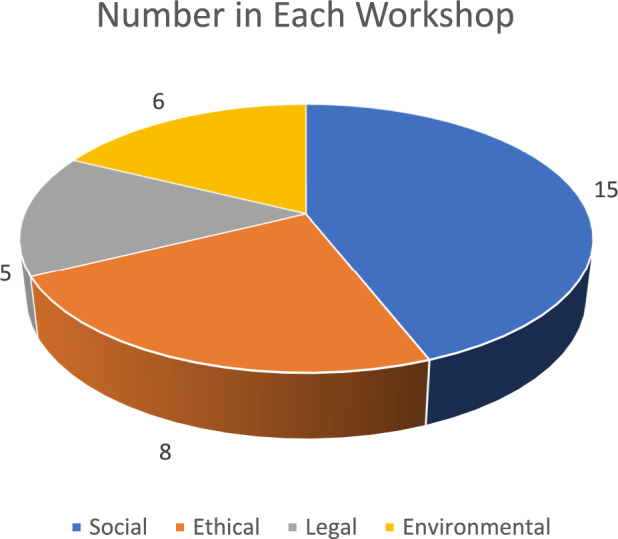
The division of participants in the four thematic workshops

This research is based on focus group discussions in four multi-stakeholder workshops as part of a conference on ethical, legal, and social aspects (ELSA) of AI use in the agri-food sector (see Appendix 1 for the workshop structure). The four workshops had different entry points (ethical, legal, social, and environmental). Still, their overall focus was on the overarching theme of the structural challenges of AI in the agri-food sector. The workshop division was a way to split up the number of workshop participants (N = 32) to have a meaningful conversation on the structural challenges of AI in agri-food. The division of the four workshops focused on specific topics rather than being too general and unclear for the participants. Also, because participants could choose which workshop to attend, it was assumed they would choose the workshop they had the most experience with (see Fig. 2).

There was much higher interest in the social workshop, with the limit of 15 participants being reached. Initially, because of the conference's focus (agri-food) and the attention AI is receiving in policymaking and law (e.g., the EU AI Act), it was thought that many attendants would be more interested in the environmental and legal workshops. On the contrary, there was a much greater interest in the social impacts of AI.^5^

**Fig. 3:**
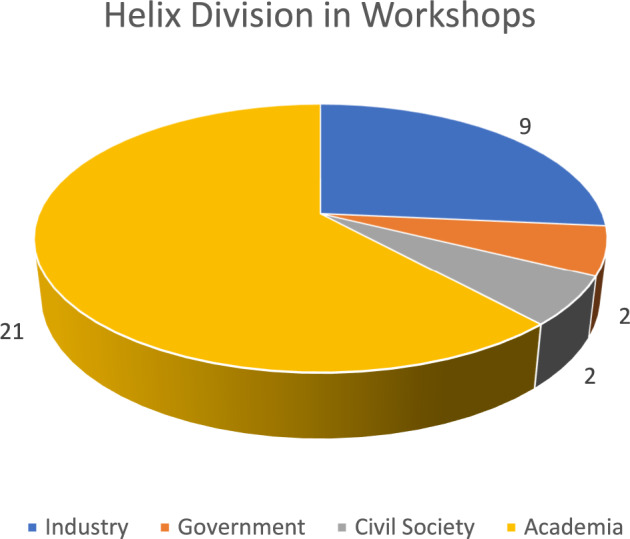
Helix division in the workshops

The workshops took place in person in the Netherlands on November 13, 2023. The four parallel workshops were each one hour long. They had diverse members from the entire quadruple helix (QH), representing policy, research, industry, and civil society (Carayannis and Campbell 2010) (with a more significant proportion from academia) (See Fig. 3).

**Fig. 4:**
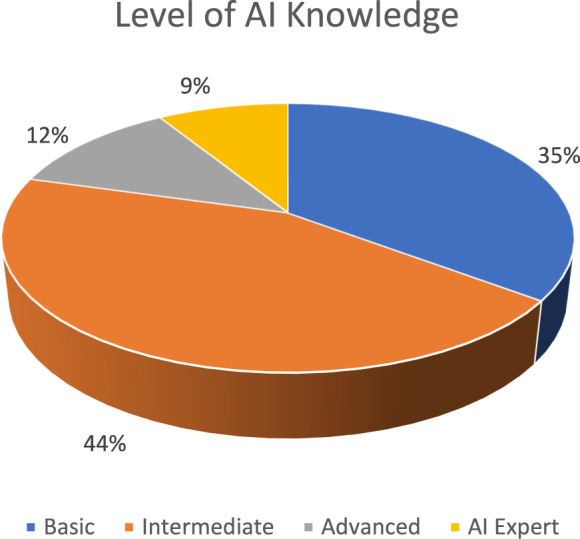
(Self-perceived) level of knowledge about ai of the participants in the workshops

The professions and backgrounds of participants were diverse, with many AI developers taking part in the workshops, as well as NGOs, governmental representatives working in the agri-food sector (ranging from technical experts on animal health, computer scientists, and legal experts), individuals working for agricultural technology providers, and social scientists. The (self-perceived) level of knowledge of AI varied among the participants. Many participants had either a (self-perceived) basic or intermediate knowledge of AI. Only 9% (3 participants) considered themselves AI experts (see Fig. 4).

Each of the four workshop facilitators gave an introductory presentation on structural challenges in AI. The participants were given hand-outs to fill in with two questions:

- What structural challenges come to your mind in the AI agri-food domain?
- How could these structural challenges be addressed?

They were asked to answer each question individually (first), followed by a plenary discussion. A dedicated note-taker documented the discussion. For consistency, all four workshops followed the same structure. The facilitators used the same PowerPoint presentation slides (with slight modifications to highlight the specific theme of each workshop) and the same workshop format.

The individual answers and the notes per workshop were entered into ATLAS.ti.22. A thematic analysis method was implemented (Braun and Clarke 2006). This analysis is ‘a method for identifying, analyzing, and reporting patterns (themes) within data. It minimally organizes and describes [the] data set in (rich) detail’ (Braun and Clarke 2006, p. 79). We implemented Braun and Clarke’s six stages of thematic analysis (Braun and Clarke 2006, p. 87): (1) data familiarization; (2) generation of codes; (3) search for themes; (4) review of themes concerning extracts that were coded; (5) definition of themes; (6) production of the paper.

First, the handouts from the workshops were digitally compiled into Excel tables with information about the QH they belonged to, their knowledge of AI, and what best represents their attitude toward AI in the agri-food sector. The facilitators familiarized themselves with their workshop data and uploaded their digitalized Excel handouts and session notes to a shared SharePoint folder. The other facilitators familiarized themselves with each other’s data.

A month later, the coordinator of the workshops organized a meeting for the co-authors to discuss the data and began incorporating the data in the qualitative analysis software tool ATLAS.ti.22. The team discussed what way to analyze the four workshops and what type of approach to take. It was decided to begin with a deductive method of coding, with the option remaining open to change this to a mixed methods approach if it was identified that inductive coding was needed later. Deductive coding offers the benefits of providing structure to data analysis, offers an efficient process among multiple varying sources, and allows a great deal of comparison among different studies. Based on the literature and what we wanted to discover from the workshops, it was decided to have three specific coding category groups (see Table 1 below).

**Table 1:** Three coding categories used to analyze the workshop data

Three coding categories
1. At which societal level is the challenge or the recommendation being described (using the micro-, meso-, or macro-levels of analysis)
2. The value(s) that underpin what the participants are referring to (using the 11 values and their definitions from Jobin et al. (2019) and Ryan and Stahl (2020)
3. What domains are significant in the challenges and solutions discussed in the workshops (using the steeple (Baruah 2020) methodology – social, technological, economic, environmental, political, legal and ethical factors – with the additional inclusion of ‘cultural’ factors)

The workshops were coded based on the level at which the topic was discussed (micro, meso, and macro). This coding was done to identify at what level the participants viewed the most significant challenges and solutions. The three societal levels were coded in two ways: First, challenges were coded based on *who was primarily impacted*. If individuals were impacted, then we coded it as a micro-challenge. If groups/businesses were impacted, it was coded as a meso-challenge; if it was a national/societal/legal system or worldview, it was coded as a macro-challenge. In contrast, the solutions were coded based on *who is primarily responsible* for initiating the change (e.g., if individuals, then it is a micro-solution; if it is businesses, groups, or communities, then it is a meso-solution; if it the government, society, or legal system, it is a macro-solution).

However, many structural challenges impact more than one level or have more than one actor responsible for initiating change (hence, we coded by identifying who was ‘primarily’ impacted or responsible). In addition, while solutions may have one very prominent actor (e.g., the national government at the macro-level), these results impact many levels (e.g., micro, meso, and macro).

The values that underpin what the participants describe were also coded to categorize what was discussed in the workshops based on 11 values found in the literature: transparency, justice and fairness, non-maleficence, responsibility, privacy, beneficence, freedom and autonomy, trust, sustainability, dignity and solidarity (Jobin et al. 2019; Ryan and Stahl 2020) (we used the definitions of these values found within these two papers).^6^

Coding values represented the values discussed in AI guidelines and principles and made these often abstract values more concrete by identifying real-world concerns in the field (Mittelstadt 2019; Veale 2020; Hagendorff 2020; Munn 2023; Bleher and Braun 2023). Analyzing the values provides a helpful way to highlight how many (traditional) value-focused topics have inherently structural implications (highlighted in our results).

The domains in the context of structural AI challenges and solutions were also analyzed to interpret what stakeholders view as the most and least essential for addressing structural challenges (see Table 2).

**Table 2:** Code groups and codes applied

Code group	Codes
Level	Micro, meso, and macro
Values	Transparency, justice and fairness, non-maleficence, responsibility, privacy, beneficence, freedom and autonomy, trust, sustainability, dignity and solidarity
Domain	Social, technological, ethical, environmental, political, legal, economic, and cultural

The third step outlined in Braun and Clark 2006 focuses on searching for specific themes within the coded texts in ATLAS.ti.22. These themes largely emerged organically during the coding process, and some emerged after the initial coding process. A benefit of the deductive coding method was that many of the themes were already pre-identified, namely, the three code groups listed in Table 1.

Steps 4 and 5 of Braun and Clark’s (2006) methodology involve analyzing and comparing the themes with the coded texts in ATLAS.ti.22. The team of five focused on each theme separately, evaluating what overarching similarities and differences emerged from the codes. This was discussed in a group meeting until a consensus was reached on the main message, how this was represented in the coded text, and if opposing or contrasting viewpoints were expressed in the coded thematic text. This analysis was done for each theme individually until there was an agreed-upon definition of each theme and what the workshop representatives expressed about this.

The workshop coordinator led Step 6 alongside the team. The results were first drafted based on the input from the meetings and the coding sessions, with several revisions and adjustments to represent best the views and thoughts expressed in the four workshops.

During the writing of the results, it was clear that specific values were coded many times and that there was a great deal of overlap between the four workshops (e.g., ‘justice and fairness’ were coded 23 times in the challenges, and 28 times in the solution, sections of the workshops). Furthermore, some information coded during the coding sessions could not be discussed in the results section because of the vagueness or brevity of the workshop participants’ comments. Lastly, as the focus of the workshops was on the structural challenges and solutions, non-structural topics arose less.^7^ The results section is a thematic analysis of the most significant topics discussed in the four workshops.

## Results

**Fig. 5:**
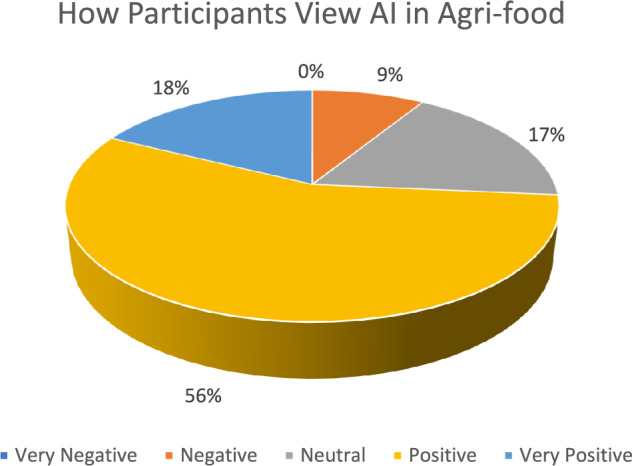
How participants viewed AI in agri-food

The first result from the workshops was how the participants viewed the development and use of AI in the agri-food sector. Most participants viewed AI in agri-food positively (56%). Only 9% viewed AI negatively, and nobody viewed it as ‘very negative’, despite several controversies and ‘existential risk’ concerns recently portrayed in the media^8^ (see Fig. 5).

**Fig. 6:**
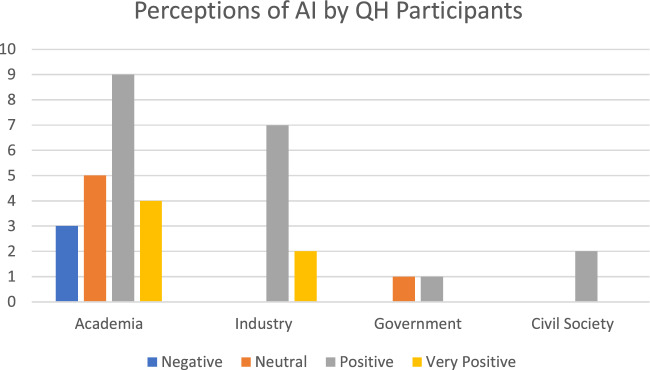
Perceptions of AI by QH participants

Industry and government representatives viewed AI as neutral to very positive, with the only negative views of AI emerging from academia (see Fig. 6).

**Fig. 7:**
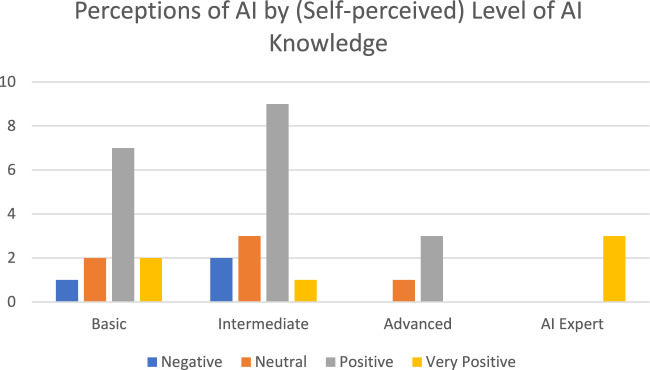
Perceptions of AI by (self-perceived) level of AI knowledge

How AI is perceived has much to do with their level of (self-perceived) experience and knowledge of AI. For example, those who viewed AI negatively possessed a basic to intermediate knowledge of AI, and those who considered themselves AI experts were unanimously positive about AI^9^ (see Fig. 7).

The participants discussed all three structural levels but emphasized macro-level and meso-level challenges and solutions (see Table 3).

**Table 3:** Workshop Analysis Results Summarized

Values most discussed	Challenges	Top-down solution	Bottom-up solution
*Macro-level*			
Justice and Fairness Freedom and Autonomy Beneficence Non-maleficence Sustainability Dignity	1. AI accessibility in the Global South 2. AI harms farming as a way of life 3. Safety concerns over an overreliance on AI 4. Instrumentalisation of animals & nature 5. Transformation of the role of the farmer	‘High taxation of big tech, monitoring of big tech’ ‘active market regulation by governments’ ‘Breaking up the companies, unions, and internationalized co-operatives’ ‘Subsidies to promote diversity of farming practices’	‘Help the co-operatives, with hundreds of members, if they know what the good practices are they could help multiply the adoption of good practices’ ‘Involve citizens in future of farming through deliberative processes’ ‘Encourage pluralism and diversity in farming practice’
*Meso-level*			
Justice and Fairness Freedom and Autonomy Beneficence Non-maleficence	1. Inequality between farm size & wealth 2. Market dominance & monopolization 3. Localized technological lock-in 4. Big Tech may create an unequal distribution of benefits from AI	‘interoperability standards’ ‘sustainability standards’ ‘data standardisation’ ‘data governance rules’	‘Farmer level—cooperations may play a role to inform and strengthen’ ‘Open source AI technologies’
*Micro-level*			
Freedom and Autonomy	1. Farmers no longer in control of their farms and lives	‘consultancy tools and legal services to address ambiguities’	‘Use the coop more—what is beneficial for the individual also helps the whole group’ ‘farmer to farmer exchange and learning as a tool for empowerment’

### Macro

While there was an even distribution among the three levels regarding challenges being discussed, the participants strongly favored macro-level discussions, often focusing on national and transnational responsibility. Specifically, the social and legal workshop participants focused more on macro-level concerns. This focus may be due to the nature of social and legal domains, which often refer to broader societal concerns, such as the need for EU or national-level legislation to mitigate certain risks. The following sections highlight macro-challenges and solutions.

#### Macro-challenges

##### Justice and fairness

The participants in the ethics, social, and legal workshops all referred to the impact of using AI (or lack thereof) in the Global South. They reflected that the Global South may not be able to gain access to AI because of high costs, or if they can, they may have to trade off their data, freedoms, or resources to gain access to it (e.g., through a type of ‘freemium’ model). This lack of access may lead to a growing disparity between wealthier nations that benefit from agri-food AI and those that cannot. Not only would the Global South be disadvantaged by the lack of affordability and access to AI, but according to the participants, AI is often only designed with specific Western values and ideals in mind. Consequently, participants stated that the Global South is only seen as a market where companies can sell their AI products and make profits, while their local, regional, and historical values are not considered.

##### Freedom and autonomy

Some participants pointed out that if farmers switch to an AI system and are later unable to maintain it, they cannot return to farming without AI. A participant in the environmental workshop stated that AI would lock the agri-food industry into a technological practice, leading to a global impact on farming, cultural practices, and knowledge and arguably creating a system that lacks resilience compared to traditional methods.

##### Non-maleficence

One assumption from the workshops was that farmers who employ AI will dominate the market because of their higher efficiency, and non-adopters will get left behind. However, a concern was what happens when AI technologies fail, and an entire sector is dependent upon it. There will be a large-scale dependency on AI systems and their adequate functioning. However, when things go wrong on a widespread scale (e.g., electrical outage or hacking), this could jeopardize the entire food system and those dependent upon it.

##### Sustainability

There was concern that AI would exacerbate sustainability and the conditions of farm animals. The use of AI would treat animals as ‘production units/machines’, where the ‘focus is on maximizing profit and minimizing cost’. In addition, AI may further disconnect farmers from the ‘land and production processes’.

##### Dignity

Another concern was the broader impact AI will have on farming as a practice, the entire sector, and what it means to be a farmer. The increase of automation of knowledge and practice may detach the farmer, which could lead to ‘the loss of ways of life, practices, and knowledge, arguably lacking in resilience compared to traditional methods’. AI may radically transform what it means to farm in future, eroding the idea of a family farm and replacing it with farming-as-a-service. They commented that the ‘mechanization of farming has already removed the farmer from the field’, and ‘AI and automation of knowledge and practice will conclude this detachment’.

#### Macro-solutions

##### Freedom and autonomy

There was an emphasis on the importance of *educating farmers* about AI and their rights regarding data-sharing. Adequate training, knowledge development, and understanding of AI and how it can benefit them should be provided. There was also a strong emphasis that governments are responsible for providing ‘education’, ‘AI literacy’, and ‘digital literacy courses’ to ensure farmers can make informed decisions. Furthermore, it was proposed that the EU should ensure the *transparency of compliant companies* with EU policy to ‘make it easy for farmers to know which service companies comply with EU/national data acts’. This indication could be done by providing a white or blacklist of which companies comply with the policy. AI companies could then be asked to demonstrate how they implement and respond to policy.

##### Beneficence

One participant stated that governments should ensure ‘benefits for farmers to share data’ while making it ‘technically easy to share data’. Others proposed that increasing the farmer’s knowledge, education, and tech awareness would allow them to know when AI would benefit them rather than solely relying on the word of AI salespeople. Governmental subsidies could ‘empower farmers to work with AI to analyze their data and create awareness among farmers’.

##### Sustainability

One of the main challenges identified by the participants was how AI would further exacerbate an already-broken livestock industry, harming farm animals and perpetuating their place as commodities. In response, some stated that a sustainability minister could be installed to address sustainability issues related to AI use in the sector, or stringent (updated) laws and animal welfare regulations to protect animals should be created.

### Meso

All workshops emphasized sectoral-specific challenges and needs. There was a lot of focus and concern around the impact emerging players in the sector would have (e.g., Big Tech) and the future of traditional actors that cannot or do not want to deploy AI (e.g., smaller farms). There was an emphasis on strengthening trustworthy relationships between farmers and co-operatives. This section highlights the meso-challenges and solutions discussed in the workshops.

#### Meso-challenges

##### Justice and fairness

The participants reflected on how unfair access to AI may arise, creating power imbalances and growing inequalities between those who can and cannot benefit from AI. They reflected that there is a potential for greater inequality between small and large farms because of the high entry costs for using AI, which would allow already wealthy farmers to benefit from AI. At the same time, ‘smaller agricultural players’ would face more pressure to adapt and may compromise their farms to keep up with their competitors who benefitted from AI.

Many were concerned about the power of large, wealthy companies such as ‘Google/MS/Amazon’, or the retail sector because ‘they have a lot of data so they have power, knowledge of the market and can have power over farmers’. These impacts may lead to market power concerns as ‘multinational agribusiness and big tech companies may dominate AI tech services in agriculture’. If a small group of providers controls AI use, it may create accessibility issues regarding who can use and benefit from AI. This could create a monopoly of data and technology providers by allowing ‘one or several companies to dominate a certain market’. Participants in the legal workshop discussed this as a ‘gatekeeper’ problem, whereby the more prominent businesses would control who has access to AI.

##### Freedom and autonomy

One participant stated that many companies only look for suitable AI solutions in their region (e.g., the US or the EU) because of the regulatory challenges with implementing AI from other regions, which restricts the freedom of farmers to choose the best solutions. They focus on adopting only local AI solutions, despite other better options being available, because they are worried that these alternatives will not abide by EU legislation. Thus, there is a lock-in toward local AI companies, not by the companies, but because of regulatory restrictions.

##### Beneficence

A participant from the legal workshop stated that small farmers may not be able to afford and benefit from AI and thus may fall behind. The participants discussed the pressure on farmers to give their data to large agribusinesses (necessary for AI), which may not necessarily benefit farmers. Many raised concerns that mostly powerful corporations developing and deploying AI would benefit from this data collection rather than the farmer.

#### Meso-solutions

##### Justice and fairness

In response to the challenge of ensuring farmers’ accessibility to AI, one participant stated that more open-source technologies would help. NGOs should participate in this and contribute to building infrastructure to facilitate open-access AI. However, a ‘balance between open access and exclusive data control’ must exist. The workshop participants stated a greater need to create sector-level cooperation and bodies to ‘play a role in informing and strengthening’ decision-making. Greater transparency would allow agri-food stakeholders to make more informed decisions when entering data-sharing agreements with retailers. In response to the lack of accessibility to and benefits of AI in the Global South (macro-challenge), guidelines were an excellent first step to help companies consider how their AI will impact ‘low to middle-income countries’. However, more efforts must be made to ‘internalize democracy’ and consider how the Global South can access and use AI.

##### Freedom and autonomy

Several participants stated that specific technical solutions would help ensure farmers’ freedom and autonomy. For example, ‘decentralized systems’, ‘federated systems’, or ‘dataspaces’ were sectoral-specific methods to help give farmers greater freedom and autonomy over their data and its use in AI.

##### Beneficence

The participants proposed making AI ‘as a service’ for farmers, allowing them to use AI when needed instead of paying a lot of money in advance to purchase it. AI as a service would allow farmers to test it before purchasing or even rent it out on a need-to-use basis. Several participants mentioned that farmer co-operatives should support farmers in benefiting from AI. They said co-operatives could ‘stimulate non-big tech solutions’ and ‘show the benefits of AI farm tools’ and help farmers adopt good data-sharing practices.

##### Non-maleficence

To avoid harm caused by AI, organizations should ensure better documentation, methodological procedures, and accountability. For example, one participant stated that ‘AI models must be optimized for detecting outliers if you compare them with expert opinions, mitigating the risk of the black box’.

### Micro

Many of the micro-level challenges and solutions came from the ethics workshop. This was unsurprising, as ethics has traditionally been more focused on micro-level challenges and solutions (as shown in the literature earlier), e.g., what someone should or should not do in a particular situation or what is a right or wrong course of action to take (typically, by individuals). Social, environmental, and legal discussions are usually at the meso- and macro-levels.

#### Micro-challenges

##### Freedom and autonomy

The workshop participants stated that if farmers invest a lot in high-tech solutions, they may find themselves trapped in a situation where they depend on AI and machine/sensor producers. With the increased use of AI, farmers may no longer be in control of their farms because AI recommendation systems dictate what to do or decisions become automated by AI technologies altogether. As one participant claimed: ‘AI is being pushed (or ‘nudged’) onto farmers as the best approach’.

#### Micro-solutions

##### Freedom and autonomy

Some gave simple ways to avoid the freedom and autonomy challenges raised by AI, such as ‘don’t use AI’ or ‘go back to small farms [in low-tech environments]’. However, these participants were computer scientists working on AI solutions and their comments were meant as jokes. However, these comments are still relevant as they could be seen as micro-level responses to the challenges of losing freedom from AI.

### Domain-level results

Lastly, our domain-level analysis (STEEPLE Baruah 2020) and cultural factors) highlighted specific foci from the workshops (see Table 4).

**Table 4:** Challenges and solutions coded by domain of relevance

Domain	Challenges Codes	Solutions Coded
Cultural	3	4
Economic	35	14
Environmental	4	3
Ethical	10	11
Legal	13	23
Political	9	13
Social	17	17
Technological	14	34

Table 4 stands out for two things: the high number of economic challenges mentioned by workshop participants and the high number of technological solutions proposed in the workshops. This result will form the basis of two discussion points in the following section.

## Discussion and conclusion

While the results were primarily context-specific to the agri-food domain, we hypothesize that many of the overarching insights gathered from the workshops may be applicable and usable in other domains. In this section, we describe four overarching insights and their relevance for other domains.

Overall, the results show that addressing structural challenges in AI is seen as essential but challenging and requires an approach that considers four elements: (1) multi-level, (2) multi-faceted, (3) interdisciplinary, and (4) polycentric governance.

### A multi-level approach is needed

The main question we posed at the beginning of our research was: How do AI professionals perceive structural challenges, and to what extent is there a need for approaches that focus beyond micro- and meso-level challenges?

The literature review revealed that the prevalence of AI ethics guidelines and frameworks is directed toward individuals or companies developing and deploying AI. AI ethics guidelines and frameworks often only focus on micro- and meso-level impacts and values, with a greater emphasis on developers and AI companies being responsible for these changes. However, our results have shown that requiring AI developers to encode values into AI systems alone is insufficient to address structural challenges.

While AI ethics guidelines and AI ethics approaches often focus on the responsibility of designers and the competencies and skills of designers to take this responsibility, this paper’s results show that many historical inequalities, structural issues, and systemic challenges are beyond their reach. This result means that while AI ethics guidelines and AI ethics frameworks are helpful, they risk overlooking more complicated, nuanced, and intersected structural challenges. Because of the complex nature of structural challenges, simply implementing a set of AI ethical guidelines or calling for more values to be built into the design of AI is not enough. While these approaches can often address many surface-level issues within AI, there is a greater need for a multi-level approach to address structural challenges (Ryan et al. 2023).

Developing a multi-level approach to the structural challenges posed by AI also highlights the need to include diverse stakeholders in discussions around AI ethics rather than solely focusing on the obligations of AI developers and companies. There are many agents, actors, relationships, and ways to address structural challenges, and many other categories of stakeholders that can help in this effort (e.g., also requiring a multi-faceted (Sect. 5.2.) and interdisciplinary (Sect. 5.3.) approach).

### A multi-faceted approach is needed

One of the findings was the emphasis among the workshop participants on technological solutions to structural challenges. An overemphasis on technological fixes is often criticized in the literature as ‘technological solutionism’ (Lindgren and Dignum 2023; Ludwig et al. 2022; Morozov 2013; Ryan and Blok 2023) or ‘technofixing’ (Dillet and Hatzisavvidou 2022; Ryan et al. 2019). Technological solutionism claims that all the world’s problems can be fixed through increased digitalization, technical innovation, and the development of high-tech solutions. These solutions often represent the interests of dominant actors while hiding the complexity or ‘wickedness’ of societal problems that involve different and even opposed viewpoints and interests (Ludwig et al. 2022).

One of the most significant insights is that while most of the 32 participants viewed AI as generally neutral to very positive (only 9% viewed it negatively), they still identified many structural challenges and concerns that should be considered (the most evident of those were seen under the headings of justice and fairness, freedom and autonomy, beneficence, non-maleficence, and sustainability).^10^This finding can be contrasted with what was illustrated earlier: industry stakeholders had a much more positive view of AI. On the other hand, representatives from academia had a much more balanced view of AI, ranging from negative to very positive. In contrast, policymakers and civil society had a neutral to positive view of AI.

This difference indicates the importance of bringing different QH groups together to discuss AI's structural challenges and ways to solve them. The industry needs more critical viewpoints on AI; otherwise, it may continue business as usual based on its optimistic assumptions about AI. On the one hand, private sector Research and Development (R&D) can concretely operationalize ELSA requirements in consumer products and services, as indicated in the emerging literature on responsible innovation in industry (Lubberink et al. 2017). On the other hand, it is beneficial for academics to discuss their concerns with the industry to ground their theories with first-hand perspectives of how AI is deployed in real-world situations rather than solely focusing on different theoretical scenarios.

Civil society organizations could also benefit from the analysis of structural challenges to serve the interests of citizens and society (see also Carayannis and Grigoroudis 2016). Their relative independence within the discussions can allow for a critical reflection on structural challenges toward citizens and society (Carayannis and Campbell 2011) and to identify social values (Popa et al. 2020). In addition, policymakers and local governmental representatives should be included to identify how to tackle structural issues at the macro-level, which became evident in our results. If dialog does not include governmental representatives, there is the risk that all responses to structural challenges will be complex to realize because change will have to rely solely on bottom-up approaches.

### An interdisciplinary approach is needed

One of the most significant insights was that economic challenges were mentioned the most out of all domains (see Table 4 earlier) despite not being explicitly emphasized during the workshop facilitators' presentations. Nor was economics a specific focus point of any of the four workshops (the focus was on social, environmental, legal, and ethical aspects).

On the one hand, this seems self-evident as most of the innovations take place in commercial or industrial settings. On the other hand, private sector R&D in AI is underrepresented in current research in responsible innovation, ELSA and AI ethics. This difference is the same in the AI ethics guidelines and AI ethics frameworks, which seem to be oriented on public sector R&D in AI. At the same time, the consideration of ELSA cannot be separated from the economic impacts and concerns surrounding AI (Ryan and Blok 2023).

It is commonly perceived that the business case logic of private sector R&D in companies (i.e., maximizing profits and serving shareholder interests) often competes with the social-ethical logic to take responsibility and serve societal goals (as is observed in the business ethics and Corporate Social Responsibility literature, see Osagie et al. 2019). While private sector R&D is primarily driven by business case logic, our results show that multiple structural challenges require a more integrative interdisciplinary approach to address structural challenges adequately rather than isolating ethical, legal, social, and environmental aspects. R&D in AI has to show how the business case can be served by considering ELSA. Business case logic primarily drives private sector R&D, while our results show that many structural challenges require a socio-ethical approach to address them. Therefore, Corporate Social Responsibility for responsible AI should be complemented by *political* responsibility for structural issues, as is argued in the business ethics literature (Tempels et al. 2017).

The literature also emphasized the importance of interdisciplinarity in AI applications (Tzachor 2022). Interdisciplinarity is required because of the ‘many interwoven and complex challenges from various disciplines that need to be addressed when developing and using AI’, which requires that ‘all the relevant disciplines need to be considered to provide a proper solution and avoid incomplete or conflicting solution alternatives’ (Ryan et al. 2023, p. 21).

Including a plurality of scientific disciplines will allow structural challenges to be better identified, explored, and addressed. This paper reflects a broader realization: responsible AI requires an interdisciplinary approach that creates sufficient space for heterogeneous viewpoints and values regarding the societal goals AI applications should serve. With heterogeneous, we refer both to the inclusion of a plurality of scientific disciplines (social sciences, humanities, computer science, and other technical sciences, such as plant, animal, and environmental sciences, depending on the domain) and non-academic actors, such as end-users, government agencies, private, non-governmental actors, and even citizens.

### A polycentric governance approach is needed

The workshop participants proposed solutions at different governance levels to address the structural challenges. This point indicates a widely felt argument that wicked and complex issues such as AI cannot be solved by the state alone (Haggart et al. 2021) nor by any other governance body. Addressing structural challenges resulting from AI requires a holistic and polycentric governance approach, combining horizontal and vertical, formal and informal governance mechanisms and regulatory interventions. Polycentric governance systems are those in which ‘political authority is dispersed to separately constituted bodies with overlapping jurisdictions that do not stand in a hierarchical relationship to each other’ (Skelcher 2005: 89).

Ensuring fair data governance for non-personal data sets is a significant structural challenge and should be addressed at the policy level (see Ryan 2019, 2020, 2022). Data ownership and rights are essential to minimizing historical and systemic power imbalances and protecting individuals’ autonomy and control.^11^ This issue can be seen in the recent European Data Act, which came into force in January 2024 (after our workshops) with clear rights, rules, and obligations for Internet of Things (IoT) generated (non-personal or personal) data. While this has positive implications for accessing and sharing non-personal data for all the IoT-generated data-driven sectors (for example, agriculture; see more in Atik 2022, 2023), more steps may be needed for greater sectoral clarification and implementation of this horizontal policy to address structural challenges related to data governance in different sectors, especially in the context of how it will be used in the context of AI.

Many of the structural challenges identified in this paper can also be related to the lack of legal certainty regarding the rules and obligations over AI applications in the farm setting. For example, the (European) AI Act will impose a legal regime that prohibits unacceptable risks and imposes certain obligations for high-risk and limited-risk categories. The proposal for the AI Liabilities Directive aims to regulate the liability rules for AI products. These horizontal legal developments may be positive from the point of view of addressing structural challenges related to AI.

However, it is essential to highlight that these regulatory initiatives, such as the Data Act, AI Act, or AI Liabilities Directive, are horizontal legal frameworks for all sectors driven by data economy and AI solutions, not explicitly catered for the nuances and idiosyncrasies of different sectors (for example, in the case presented in this paper—agri-food). Therefore, some remaining sectoral issues may not be adequately addressed and need complementary governance arrangements (see Atik 2023, for an agri-food sectoral analysis of the Data Act).

Farmer co-operatives, civil society organizations, and NGOs must become more involved and work with farmers to empower farming communities and develop local knowledge on using and benefitting from AI. This argument is in line with (Dara et al. 2022), who show that through farmer cooperatives, farmers can get more involved in how AI is developed and have a say in how AI can benefit them and the sector (Dara et al. 2022). Education and digital literacy skills were a key focus of the workshops to help reduce structural challenges caused by AI (see also Perc et al. 2019), and the appropriate level to address this, according to participants, was the NGO and cooperative (i.e., sub-national level).

Identifying the underlying reasons for structural challenges is a critical precondition for better designing sectoral policies and rules. In this context, a polycentric governance lens can help understand how specific macro-structures cause the systemic ordering of a policy field such as AI.

### Limitations and further research

One of the limitations of our research was the (relatively) small sample size (N = 32). There were also only a few participants from civil society and the government. While many civil society and governmental representatives were invited, most could not attend. Another potential bias or limitation is that the sample was within the Netherlands. There may have also been an overrepresentation of computer scientists, which resulted in the significant emphasis on technological solutions discussed earlier. Lastly, people and citizen representatives were not explicitly included in the workshops.

Further research may be conducted into other country-specific focuses on structural AI challenges or larger geographical samples (e.g., Europe or other world regions). Further research should also be conducted on structural challenges with laypeople impacted by and through AI. Because we make many grander (more general) claims about the requirements to address structural challenges based on our context-specific results, further research must be conducted in other sectors and domains. Our results could be used to identify commonalities and differences between other sectors and regions.

## Data Availability

No formal ‘data’ were used for this paper. The paper is based on four workshops, where the information retrieved was initially taken down as notes to recall what was said during the workshops. These data were further expanded upon and developed in the paper so all of the data that were used/are available are included in the paper. The notes taken from the workshop include sensitive data about the workshop participants, and we do not want to include this as supplementary files because the information is sensitive, and we did not ask the participants’ consent to share these raw files externally.

## Appendix

Before the event, the facilitators held several meetings to ensure consistency among the presentations, the workshop facilitation, and the notetaking from the workshops (this was done by a separate note-taker so that the facilitator could concentrate on facilitating the workshop). Each workshop was divided into approximately 3 sections (Sect. 1 was the presentation of the topic by the facilitator, Sect. 2 was the identification of structural challenges on the given workshop topic by the participants, and Sect. 3 was the discussion of solutions to respond to the challenges discussed in Sect. 2).

Section 1 of the workshop consisted of a presentation created by the workshop coordinator to ensure consistency among all workshop facilitators. Sections 2 and 3 began with the participants writing down their views in a text-box hand-out distributed by the workshop facilitator. The purpose of this was to obtain written data from participants on their views and to ensure that everyone’s views would be read after the workshop (with the awareness that some people do not like discussions/speaking in workshops).

The hand-outs contained three sections for preliminary information collection: which of the four helices did the participant belong to (options: government, academia, industry, and civil society); what was their level of AI knowledge (options: basic, intermediate, advanced, to AI expert); and what best represents their attitude toward AI development in the agri-food sector (options: very negative, negative, neutral, positive, very positive).

Following these opening questions, there were three text boxes with the questions above them:

1. ‘When you think about the role of AI in sustainable agri-food systems, what structural challenges come to your mind on this topic? And why? Is this challenge difficult to solve? Why?’
2. ‘When you think about the structural challenges just discussed, what kind of recommendations or solutions can you think of to respond to these challenges? And why?’
3. ‘Do you have any more thoughts/additions?’

Participants were given 5 min to write down their thoughts on the hand-out for question 1 above, followed by 15 min each to discuss the structural challenges of AI in the agri-food sector. The same was done for question 2 above on the solutions on addressing the structural challenges of AI in the agri-food sector.

## Footnotes

1 Other examples include AI alienation (Wogu et al. 2018; Haga 2022; Engel 2019), data subjectivation (Lippold 2018; Matzner 2018), AI instrumentalism (Gill 2020), datafication (Blok 2023; Veraart et al. 2023), anthropocentrism (Stellinga and Blok 2024; Hagendorff 2022), techno-solutionism (Saetra 2023; Brevini 2023), de-humanization (Fritts and Cabrera 2021; Rhee 2018)).

2 When we refer to AI professionals, we simply refer to the diverse group of participants involved in the workshop who were either working on the technical aspects of AI (e.g., developers, computer scientists, technicians, etc.) and/or those working on the non-technical impacts of AI (e.g., NGO representatives, governmental representatives, ethicists, legal experts, and social scientists). All participants attending the conference, and thus workshops, were working on the topic of AI in agri-food.

3 In the AI literature, the term 'AI system' is often interchangeable with others like 'algorithmic systems' (Crawford & Calo 2016), 'automated decision-making systems' (AlgorithmWatch 2019), 'AI-based systems' (Sadek et al. 2023), or 'autonomous/intelligent systems' (IEEE SA 2020). While there might be subtle differences, for simplicity, we consistently use the term 'AI system' in this paper, following the definitions of OECD (2022) and the European Commission (2021).

4 Ethics generally asks questions such as 'What is a good action?', 'What is justice?', etc. AI Ethics is generally viewed as an example of applied ethics, about 'what we are obligated (or permitted) to do in a real-life and often new situation.' Specifically, this 'focuses on the normative issues raised by the design, development, implementation and use of AI.' 'Ethical AI is used to indicate the development, deployment and use of AI that ensures compliance with ethical norms, including fundamental rights as special moral entitlements, ethical principles and related core values' (European Commission 2020, 37).

5 Power is such a core and central theme within the analysis of systemic challenges in society, so this was already a confirmation that many stakeholders working in this area considered structural challenges in this sector or at least wanted to hear more about them.

6 In relation to the definition of privacy, it can be seen as a value to uphold or a right to protect (Jobin et al. 2019) through data protection, cybersecurity, de-identification, encryption, and other means (Ryan and Stahl, 2020). However, neither article makes explicit what they mean by privacy, so we used several legal and human rights definitions as our basis for coding examples of privacy in the workshops.

7 The micro-level challenges that will be discussed in Sect. 4.2. are commonly discussed in AI ethics literature and outlined in AI ethics guidelines (e.g., privacy, security, and transparency). They can be both structural or non-structural in nature, depending on how they are contextualized. In the workshops, there were also many non-structural issues discussed related to micro- and meso-level challenges and solutions, such as: identifying who is responsible/liable when AI makes mistakes, how to ensure transparency of AI (environmental workshop), debiasing datasets (environmental workshop), and data security (social workshop). Therefore, we focused on the main structural-level challenges discussed in the workshops, with less of a focus on the non-structural issues, as this is the objective of the paper.

8 The participants' knowledge of AI is a possible reason for this – they realize the current state (and limitations) of AI and do not buy into the hype of superintelligence or singularity.

9 This could be explained by the fact that these experts have worked on AI solutions for many years and have developed positive attitudes toward their research.

10 Besides sustainability, these four values correlate with Beauchamp and Childress’ four principles of biomedical ethics (autonomy, non-maleficence, beneficence and justice) (1979). Sustainability has become a significant value in society in recent years, and this is also represented in the literature on AI in the agri-food sector (Ryan 2022; Ryan et al. 2023). Therefore, it was unsurprising that it was also discussed during the workshops (mainly because one of the workshops focused on the environment and sustainability, which has an essential role in the agri-food sector).

11 Some participants reflected on the possibility of the farmer being a ‘co-owner’ with the agricultural technology provider. Still, there are ambiguities about data rights. While in some circumstances, the farmer remains the owner of the raw data, they still have very little control over how this data is processed and the value derived from it.
